# Design and Development of the “*POD Adventures*” Smartphone Game: A Blended Problem-Solving Intervention for Adolescent Mental Health in India

**DOI:** 10.3389/fpubh.2019.00238

**Published:** 2019-08-23

**Authors:** Pattie P. Gonsalves, Eleanor S. Hodgson, Avinash Kumar, Tiara Aurora, Yash Chandak, Rhea Sharma, Daniel Michelson, Vikram Patel

**Affiliations:** ^1^Sangath, New Delhi, India; ^2^Quicksand Design Studio, New Delhi, India; ^3^School of Psychology, University of Sussex, Brighton, United Kingdom; ^4^Department of Global Health and Social Medicine, Harvard Medical School, Boston, MA, United States; ^5^Harvard TH Chan School of Public Health, Boston, MA, United States

**Keywords:** mental health, adolescents, LMIC, gamification, problem solving, blended, smartphone applications (apps), digital

## Abstract

**Introduction:** Digital technology platforms offer unparalleled opportunities to reach vulnerable adolescents at scale and overcome many barriers that exist around conventional service provision. This paper describes the design and development of *POD Adventures*, a blended problem-solving game-based intervention for adolescents with or at risk of anxiety, depression and conduct difficulties in India. This intervention was developed as part of the PRemIum for ADolEscents (PRIDE) research programme, which aims to establish a suite of transdiagnostic psychological interventions organized around a stepped care system in Indian secondary schools.

**Methods and Materials:** Intervention development followed a person-centered approach consisting of four iterative activities: (i) review of recent context-specific evidence on mental health needs and preferences for the target population of school-going Indian adolescents, including a multiple stakeholder analysis of school counseling priorities and pilot studies of a brief problem-solving intervention; (ii) new focus group discussions with *N* = 46 student participants and *N* = 8 service providers; (iii) co-design workshops with *N* = 22 student participants and *N* = 8 service providers; and (iv) user-testing with *N* = 50 student participants. Participants were aged 12–17 years and recruited from local schools in New Delhi and Goa, including a subgroup with self-identified mental health needs (*N* = 6).

**Results:** Formative data from existing primary sources, new focus groups and co-design workshops supported a blended format for delivering a brief problem-solving intervention, with counselors supporting use of a game-based app on “offline” smartphones. User-testing with prototypes identified a need for simplification of language, use of concrete examples of concepts and practice elements to enhance engagement. There were also indications that participants most valued relatability and interactivity within real-world stories with judicious support from an in-app guide. The final prototype comprised a set of interactive and gamified vignettes and a structured set of problem-solving questions to consolidate and generalize learning while encouraging real-world application.

**Discussion:** Findings shaped the design of *POD Adventures* and its delivery as an open-access blended intervention for secondary school students with a felt need for psychological support, consistent with an early intervention paradigm. A randomized controlled trial is planned to evaluate processes and impacts of *POD Adventures* when delivered for help-seeking students in low-resource school settings.

## Introduction

Mental health problems account for over a third of the total burden of disease in adolescents (1), withs depressive, anxiety and conduct disorders together accounting for over 75% of this burden (2). India is home to the world's largest population of adolescents, placing the country at the center of global efforts to improve mental health care for this age group. At the same time, fewer than 10% of young Indians have access to formal mental health services (3). The negative impacts of adolescent mental problems are starkly reflected in the strong association between poor mental health and long-term social disability, while suicide is the leading cause of death for 15–24 year olds in India (4–6).

The large mental health care gap in India and other low-and middle-income countries (LMICs) coincides with a rapid boom in telecommunications and internet access. Evidence from diverse low-resource settings shows that young people aged 10–24 years adopt new technologies and use mobile devices and the internet more frequently than individuals from older age groups, including for the purpose of accessing health-related information (7). India in particular has 90% mobile phone penetration, more than 225 million smartphone subscribers, and rapidly increasing rates of internet and social media usage (8). Despite varied access and gaps in connectivity, especially in rural areas, digital technology platforms offer unparalleled opportunities to reach vulnerable adolescents at scale and overcome many of the barriers that exist around conventional service provision (7, 9).

Since the initial description of “e-health” by Eysenbach (10), the nature of such digital provision has evolved considerably. Static content delivered via personal computers (PCs) or laptops has been superseded by more advanced technology that allows for greater functionality and adaptability. This increasingly involves delivery of interventions through applications (“apps”) designed for smartphones and other wearable digital devices (11). There are now more than 10,000 publicly available mental health apps (12, 13), of which a growing number make use of “serious games,” i.e., games that are designed to educate, train, or change behavior as they entertain players (14). However, few serious games to date have been tested and reported in the scientific literature; the available reports are based almost entirely on desktop-computer formats rather than mobile apps (15).

Meta-analyses and systematic reviews of mental health apps for adolescent populations highlight promising findings for feasibility and acceptability (16–20). Game-based approaches offer specific characteristics that can help make learning more meaningful, engaging, visual, and interactive (15). However, evidence of efficacy remains limited, especially for apps evaluated in LMICs and for those focused specifically on adolescent mental health (15, 17, 18). A recent review of digital technology for treating and preventing mental health problems in LMICs identified 49 studies, including only four studies with adolescents or youth (7).

In this paper, we describe the design of *POD Adventures*, a blended problem-solving game-based intervention for adolescents with or at risk of anxiety, depression and conduct difficulties in India. *POD Adventures* was developed as part of the PRemIum for ADolEscents (PRIDE) research programme (2016–2020), which aims to establish a suite of transdiagnostic psychological interventions organized around a stepped care system in Indian secondary schools (21). PRIDE has also developed and evaluated a counselor-led problem-solving intervention in secondary schools across New Delhi and Goa in India. The counselor-led problem-solving intervention and associated sensitization activities (aimed at generating self-referrals) have been evaluated in randomized controlled trials at the New Delhi site (Parikh et al., under review).

*POD Adventures* was originally conceptualized as a digital counterpart to the PRIDE counselor-led problem-solving intervention, with both aiming to enhance a participant's ability to cope with stressors and thereby prevent/improve mental health problems (22), in line with stress-coping theory (23). In terms of delivery, *POD Adventures* follows a blended approach that deploys low-intensity human support in tandem with a smartphone-delivered game, potentially reducing the resource demands of conventional face-to-face counseling. Thus, we aimed to develop an intervention specification that could bridge the gap between the high prevalence of anxiety, depression and conduct difficulties and low capacity of the public mental health system in India and potentially other similar settings (24).

## Materials and Methods

### Research Design

An iterative approach was taken to intervention design following established design guidelines including the person-centered approach by Yardley et al. (25) and World Health Organization (WHO) guidelines on monitoring and evaluating digital health interventions (26). The research design also incorporated insights from linked research activities related to the development and evaluation of PRIDE's counselor-led problem-solving intervention.

The primary objectives of the current study were to:

understand access to and appropriateness of a digital intervention for adolescents in schools in India

identify features to enhance engagement with, the problem-solving content of the intervention

identify features to optimize the developmental and cultural acceptability and usability of the intervention

identify features to optimize contextual acceptability and feasibility of intervention delivery

### Setting

Intervention development was initiated in July 2017 at sites in New Delhi (north India) and Goa (southern India). These sites were selected for the current study as services, staff and school relationships were pre-existing in these two regions. Goa and Delhi are among India's most highly urbanized states and offered opportunities to evaluate the roll out of a technology-enabled intervention. Primary data collection was conducted in two same-sex government-run, Hindi medium secondary schools in New Delhi (one all-girls and one all-boys) and six co-educational government-aided secondary schools in Goa.

### Ethical Approvals

Approvals for PRIDE's formative research activities, including the current study, were obtained from the Indian Council of Medical Research (ICMR) and institutional review boards of Sangath (the implementing organization in India) and London School of Hygiene and Tropical Medicine (a collaborating academic partner in the UK). Local approvals were also obtained from the relevant school boards in New Delhi and Goa. Informed written assent and consent was obtained from participating adolescents and their parents/guardians. Detailed information sheets providing information about the PRIDE programme, focus group discussion and co-design workshops were included, along with assent/consent forms that were developed in English, Hindi, and Konkani (the local languages). Participants were provided with certificates of participation for their time and contributions.

### Data Collection

Intervention design consisted of four main activities: (i) review of previous formative work; (ii) focus group discussions (FGDs); (iii) co-design workshops; and (iv) user-testing. Insights from (i), (ii), and (iii) were used to define the intervention “guiding principles” (25) leading to the specification of an initial prototype. Insights from (iv) helped to refine iterative prototypes following from emergent findings on feasibility, acceptability, and usability.

### Previous Formative Research

A review of the previous formative research consisting of qualitative studies [(27), Parikh et al., under review] and pilot studies (21) contributed to intervention planning activities. Two qualitative studies were conducted that consisted of focus group discussions with multiple stakeholders including school-going adolescents from low-and middle-income communities, parents, teachers, school counselors, and service providers at the two research sites in New Delhi and Goa with. An iterative phased approach was then used to model and then test successive prototypes of the intervention in two linked pilot studies (21) at the New Delhi site using a prospective cohort design. The studies evaluated the acceptability and feasibility of the intervention delivery and potential for impact in order to refine the intervention. An ongoing randomized controlled trial is being conducted to evaluate the effectiveness of the intervention (Parikh et al., under review).

### Focus Group Discussions

#### Participants

Forty-six adolescents (*n* = 23 in Delhi; *n* = 23 in Goa; 21 female: 25 male) participated in six FGDs (one per school at four schools in Goa; one per school at two schools in Delhi) (see Table 1). Participants were drawn from grades 8 to 11 and ranged in age from 12 to 16 years (mean 14.0), with recruitment carried out through a combination of classroom announcements and teacher nominations. To capture different perspectives, we tried to approximate equal quotas with regards to age and gender. No specific eligibility criteria were used to select adolescents on the basis of current/prior experiences of mental health problems, although participants were required to be proficient in English, Hindi, or Konkani, and to provide informed assent and parental consent. In addition, we conducted two FGDs at each site drawing on a group of eight service providers (*n* = 3 in Delhi; *n* = 5 in Goa) with experience of delivering the PRIDE counselor-led problem-solving intervention in the collaborating schools [see (21) for further details of service providers].

**Table 1:** Participant characteristics FGDs and co-design workshops.

**Activity**	**Location**	**Participants (*N*)**	**Number of sessions**	**Age range (years)**	**Female:Male (*N*)**
Focus group discussions (FGDs)	Goa	Students (23)	4	12–16	11:12
	Delhi	Students (23)	2	14 – 16	10:13
	Goa	Service providers (5)	2	–	5:0
	Delhi	Service providers (3)	2	–	3:0
Co-design workshops	Goa	Students (9)	1	14–15	4:5
	Delhi	Students (13)	1	13	0:13
	Goa	Service providers (5)	1	26–35	7:5

#### Topics

Participant FGDs consisted of questions about the use and preferences for different digital media; access to smartphones; and nature of use, location of use and experience playing games and apps. Service provider FGDs consisted of questions regarding the appropriateness of a digital mental health intervention in school settings. This included reflections on successes and challenges faced in delivering a brief problem-solving intervention in a conventional face-to-face individual format. Each FGD lasted 60–90 min and was conducted jointly by the first author and representatives from a partner design agency. FGDs were audio-recorded and documented through written notes and photographs of group work.

### Co-design Workshops

#### Participants

Twenty-two participants (*n* = 13 in Delhi; *n* = 9 in Goa; 4 female: 18 male) participated in one co-design workshop each conducted at the participating schools at the two research sites (see Table 1). The mean age of adolescents was 14 years (range 13.0–15.0 years) from grades 8 to 10. Eligibility criteria and recruitment procedures were the same as in the FGDs. The same group of eight service providers participated in one design workshop at each site.

#### Topics

Co-design workshop activities with participants included: (i) exploring a selection of popular games and apps such as Temple Run, Candy Crush, Tekken, Horizon Chase, and *Kaun Banega Crorepati?* (an Indian version of *Who Wants to be a Millionaire?*) on smartphones; (ii) story building to create personas and problem scenarios; (iii) paper prototyping of game components and characters; and (iv) discussion about prototype ideas presented by participants. Workshops with providers focused on: (i) prototype contents; (ii) identifying narratives for the game and how to emphasize the key problem-solving features; and (iii) suggestions for making the game easier to understand and navigate for users. Each workshop lasted 90–120 min and was conducted jointly by the first author and team members from the partner design agency. Workshops were documented through photographs of group work, and were audio-recorded and transcribed.

### User-Testing

#### Participants

Fifty participants (28 female: 22 male) from six schools took part in 22 user-testing sessions over 9 months at the Goa site (see Table 2). The mean age of participants was 14.5 years (range 12–17 years) from grades 8 to 12. Participants were recruited in three ways: (i) classroom announcements; (ii) teacher referral of participants representing a mix of age and gender within their classes; and (iii) participants with prior experience of school counseling who were invited to volunteer.

**Table 2:** Participant characteristics—user testing.

**Student group**	**Participants (*N*)**	**Age (range in years)**	**Gender Female(*N*): Male (*N*)**	**Source of recruitment**	**Number of sessions**
School 1	6	13–15 years	4:2	Teacher	1
School 2	6	12–15 years	3:3	Teacher	4
School 3	8	12–15 years	4:4	Volunteers	7
School 4	12	15–17 years	6:6	Teacher	2
School 5	12	12–15 years	8:4	Teacher	5
School 6^*^	6	15–17 years	3:3	Teacher	3
Total	50	12–17 years	28:22	–	22

* *Prior experience of PRIDE face to face counseling program*.

#### Topics

User-testing sessions consisted of researcher-supported gameplay, paper-prototyping and discussion-based feedback to provide insights on intervention acceptability and usability. Participants were asked about their views about the gameplay contents and concepts; characters; gamified components such as rewards, quizzes, missions, etc., as well as on ease of use, game bugs and points in the game when external support was required. Feedback was also sought on human support and intervention delivery options in schools such as small group delivery. Gameplay was observed and notes taken by supervising researchers. Discussions were documented through photographs, and were audio-recorded and transcribed.

### Data Analysis

**Figure 1:**
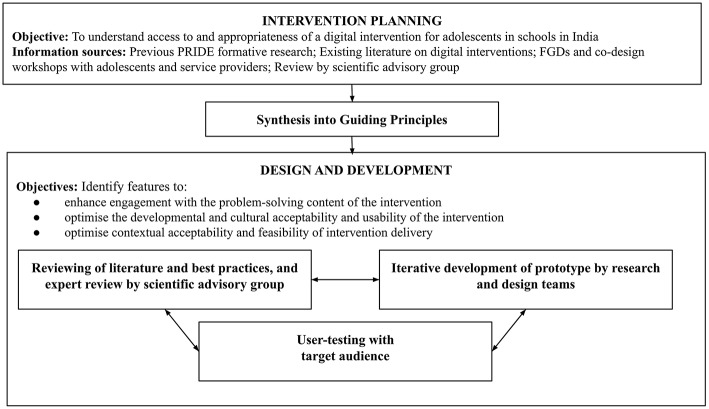
Research design process.

Analysis followed an iterative and recursive process which was led by an intervention working group (PPG, EH, RS) with oversight from the senior investigators (DM and VP) and an independent Scientific Advisory Group **(**see Acknowledgments). Insights from the parallel PRIDE formative research activities [(21), (27), Parikh et al., under review], new FGDs, co-design workshops and user-testing were analyzed and triangulated around the four research objectives using an integrated inductive-deductive approach to content analysis and through thematic and mapping techniques (see Figure 1) (28). This resulted in a set of “guiding principles” (25) that summarized the intervention objectives and distinctive features necessary to meet these objectives, in alignment with key information about the target population and its context. During prototype development, the formative data sources were revisited regularly and reviewed alongside the wider literature on digital interventions for adolescent mental health, insights from user-testing and from implementation of the counselor-led intervention that was being conducted in parallel.

## Results

### Accessibility and Appropriateness of Digital Interventions

#### Context of Self-Help

Self-help was suggested early in the formative PRIDE research (21) as a scalable format however consultations with stakeholders (Parikh et al., under review) and service provider FGDs highlighted that “self-help” was not a culturally-congruent concept for most Indian adolescents. Service provider and participant FGDs revealed norms around seeking or receiving direct instruction from parents, teachers and other elders. Both groups felt that support from a counselor would be necessary for an intervention to be effective. Service providers also reported that students they had previously provided counseling services to had difficulties understanding abstract concepts. This was linked to the emphasis on rote learning over critical thinking in the Indian schooling system. The game was therefore designed to incorporate a combination of teaching methods including direct instruction, modeling and practice to accommodate different learning styles and to emphasize self-efficacy (29–31).

#### Accessibility and Compatibility of Technology

Most participants in the FGDs reported having easy access to smartphones, albeit through devices that usually belonged to a parent (mainly fathers). The primary use was to play offline games, while internet access was restricted by limited data packs. Participants navigated a variety of digital and app-based games with minimal help, lending support to the acceptability and usability of a gamified format. Formative work (Parikh et al., under review) revealed that digital interventions were broadly appealing to adolescents. More specifically, participants and service providers felt that a digital delivery format might offer more opportunities for personalization and engagement in the context of self-help, thereby addressing limitations raised about the usefulness of this modality. Moreover, FGDs indicated that typing into a smartphone may be preferred by many participants to writing on paper. On the other hand, service providers highlighted potential concerns arising from parents and teachers regarding the amount of time that adolescents spend on phones. Based on these learnings, the decision was taken to restrict delivery to counselor-supported use of smartphones during dedicated school-based sessions, and for functionality to be independent of internet access (i.e., work offline).

#### Ensuring Privacy

The importance of providing participants with clear assurances about privacy and confidentiality emerged strongly in the preliminary formative work (Parikh et al., under review). In line with best practice guidelines for other mental health apps (19), an adolescent-friendly privacy policy was drafted in simple language and included in the game prototype. This policy contained details about how information would be collected, used and stored and under what circumstances it would be shared (i.e., in the case of risk to self or others).

#### Shift to Open-Access Delivery

Piloting of the PRIDE counselor-led problem-solving intervention generated high demand, including a majority of self-referred adolescents who scored below thresholds for clinical caseness (21). In light of this finding, and supported by feedback from participant FGDs and user-testing that problem solving would be a useful and valued skill for all adolescents (not just those experiencing ongoing mental health difficulties), *POD Adventures* was re-conceptualized as an open-access intervention for adolescents with a felt need for psychological support. This decision was consistent with evidence for problem solving as an effective prevention strategy for adolescents with emotional problems (32–34) as well as wider evidence and emerging service models for early intervention for adolescent mental health problems (35, 36).

### Problem-Solving Contents

#### Format and Structure

**Figure 2:**
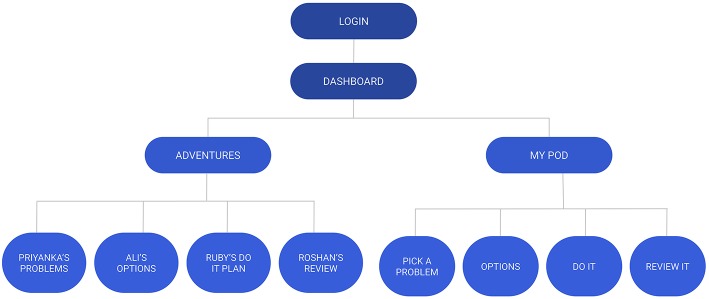
Intervention prototype structure.

**Figure 3:**
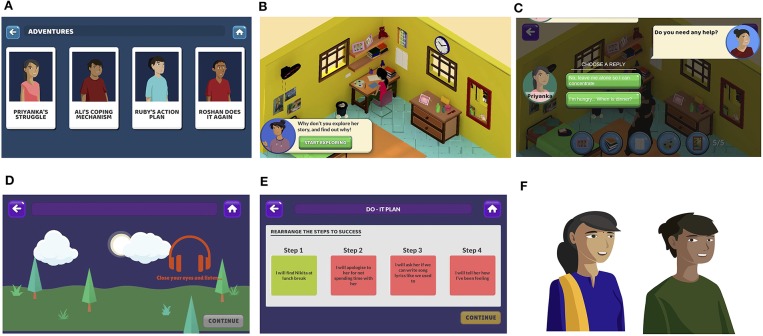
Adventures section screenshots. **(A)** Adventures home screen, **(B)** character and environment scene, **(C)** interactive conversation, **(D) “**safe place**”** guided imagery emotion regulation exercise, **(E)** mini-game to create “Do-It” plan for a character, and **(F)** examples of characters.

**Figure 4:**
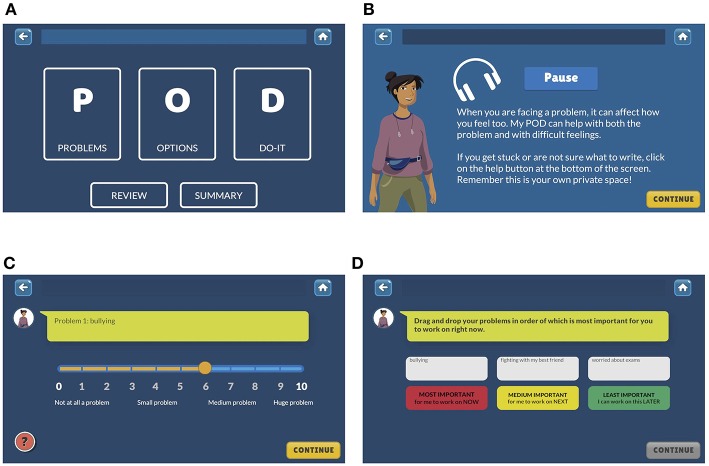
My-POD section screenshots. **(A)** My POD homescreen, **(B)** instruction screen with audio, **(C)** problem rating screen, and **(D)** problem prioritization screen.

Piloting of the counselor-led intervention supported a simplified three step problem-solving procedure with the memorable acronym, “POD” (identifying “Problems,” generating “Options,” and creating a “Do it” plan) (21) and this was adopted in development of the game. Also consistent with the counselor-led intervention that used printed comic booklets to illustrate problem solving, both participants and service providers suggested that a narrative format would be optimal for the game. The prototype structure comprised two main sections, adventures and My-POD (see Figure 2), with navigation within and between sections supported by a guide character. The adventures section contained short vignette-based stories exploring different adolescent characters and their problem-solving journeys while emphasizing the steps of problem solving (see Figure 3). The My-POD section was designed to help generalize and consolidate learning of the previous steps through real-world application. This section presented participants with a series of forced-choice and open-text questions in which they were guided through the steps of problem solving for their own problems (see Figure 4).

#### Common Problems and Stress Reactions

PRIDE formative work (27), FGDs and co-design workshops identified common social stressors including academic pressure, bullying, difficulties in romantic relationships, negotiating parental and peer influences, and exposure to violence and other threats to personal safety. Stress reactions were commonly described using the catch-all term “tension,” while more specific references to anger, rumination, and loss of concentration were also prominent in adolescents' narratives (27). The four character-based vignettes in the game were scripted to reflect some of these common problems and stress reactions. During user-testing, participants consistently reported that the vignettes were relatable but that they would like more character details and more vignettes. In response, game scripts were revised to include more detail for the central characters (e.g., more “thought bubbles” which shared a character's' thoughts and feelings; see Figure 3). However, due to the brief nature of the intervention a decision was made to limit the number of vignettes to four.

#### Concepts

User-testing sessions highlighted that while specific problem-solving behaviors were learned with relative ease, conceptual learning intended to address problem orientation was limited. My-POD questions were revised to provide concrete examples wherever possible (for example, the Options section provided the instruction, “Write down some good things about this option” and was supplemented by help text saying, “For example, a good thing might be that the option would be easy for you to do, or that it would make a BIG difference to your problem”). Additionally, an introductory animated video explaining the overarching stress-coping model and concepts of problem solving was incorporated into the game opening. These concepts were reinforced repeatedly at key points throughout the game.

Early versions of My-POD contained a high proportion of incomplete or inaccurately filled fields. Participants reported that some of the activities in this section, particularly related to option generation and selection (for example, weighing pros and cons) were difficult. Instruction screens containing text and audio information were added (see Figure 4), free-text questions were supplemented with sliding scale responses (see Figure 4), and a summary section was added to easily view action planning progress. These enhancements resulted in greatly improved completion of this section.

Participants also expressed a desire to access more directive tips and specific suggestions about ways to solve problems. In response, an interactive “Options Bank” was designed around different life domains (for example, problems faced at school, at home, in their neighborhood, emotion-focused problems, etc.).

#### Emotion Regulation Activities

Formative PRIDE work (27) indicated adolescents employed avoidance more frequently than active coping when faced with stressful situations. A key component of effective treatment therefore was in helping participants understand and manage emotions (Parikh et al., under review), also supported by findings from the service provider FGDs. To address this, emotion regulation exercises were embedded in the Adventures section of the game. These exercises were designed based on an abbreviated form of Nezu et al. “Stop, Slow down, Think, Act” method (22), and included guided imagery, breathing, and muscle relaxation exercises (see Figure 3). In user-testing, participants reported that the early iterations of these activities were too long and these were subsequently revised to be kept under 2-min in order to minimize boredom and disengagement.

### Developmental and Cultural Acceptability

#### Media Preferences

During FGDs and co-design workshops, participants expressed a preference for games with stories set in varied real-world environments and offering choices which could be explored. In response, gameplay was organized around a set of interactive vignettes located in age-appropriate settings (for example, at school, home, playground, etc.; see Figure 3). The three-dimensional immersive design of the game was based on photographs taken by game developers at the schools and surrounding localities and the design aesthetic was informed by participants' media preferences. Special care was taken to ensure a cast of culturally relatable characters representing a mix of genders, ages, body shapes, social classes, and common names.

The overall tone of the game was designed to be non-directive with multiple features providing user-choice (37, 38). Some differences in media preferences were noted between girls and boys, such that boys appeared to prefer immersive games and girls favored puzzle-platform games. The prototype was therefore designed to include both narrative and puzzle elements. In user-testing, participants strongly endorsed elements of user-control and interactive content and expressed a desire for more features of this type. In response, additional user-controlled decision points such as options to select conversational responses were added (see Figure 3). The game was designed to be available in the two most commonly spoken languages (English and Hindi) and a vernacular language spoken at the Goa site (Konkani).

#### Literacy

Formative PRIDE research revealed widespread literacy difficulties among students in Government-run and Government-aided schools, which limited the use of text-heavy printed problem-solving materials (21). User-testing also highlighted the need for more concrete and specific language, particularly around problem-solving concepts. This led to a number of changes in phrasing, for example, “Do you think you will face any challenges?” was revised to “What might stop you?” The lexicon of emotion words was also reduced, and paired with illustrated “smileys” to aid comprehension.

#### Gamification

Participants co-design workshops highlighted several gameplay features with the potential to enhance motivation. In particular, participants created mock games that included quizzes, obstacles and fighting elements, reflecting preferences for elements of competition and rewards. Consequently, vignettes were designed to include “missions” in which users completed a series of tasks in order to progress through the vignette (for example, clicking on objects in a room to discover a character's worries represented by that object). “Mini-games” were also incorporated to model, practice, and reinforce specific problem-solving behaviors (for example, rearranging the steps to make a Do-It plan for a character; see Figure 3). Quizzes were also incorporated to assess recall at the end of every vignette. During user-testing, the mini-games were among the most popular features. However, virtual “badges,” originally introduced to reward completion of mini-games, were poorly understood and generally disliked in favor of more targeted verbal encouragement. In response, the badges were replaced with motivational feedback from the guide character and points awarded for correct quiz responses.

### Intervention Delivery

#### In-Game Support

Participants endorsed both relational and instructional functions provided by face-to-face contacts with school counselors (Parikh et al., under review). Service provider FGDs suggested that a guide character within the game could provide some of these functions. The game guide character was designed to provide instructions, information (psychoeducation and teaching the problem-solving method) and support (praise, encouragement, and motivational statements) throughout the game. They were conceptualized as a warm and knowledgeable older peer, intended to enhance dialogue support and social support features in line with persuasive systems design (38, 39). Contingent responses from the guide were programmed wherever possible. For instance, if participants rated their mood as negative, the guide would respond with a sympathetic and encouraging message. User-testing indicated that the guide character was generally liked by participants. However, users of early prototypes felt that the guide appeared too often and that some of their comments were unnecessary. The guide feature was subsequently revised to appear less frequently during the Adventures section and their script was revised to focus on reinforcing key learning about the problem-solving method. During user-testing some participants reported that they felt supported by the game itself (rather than specifically by the guide) and that they enjoyed and felt safe in sharing their problem within the game.

#### Human Support

Most spontaneous requests for help during user-testing were made when participants required troubleshooting of game mechanics (e.g., which button to press next). Difficulties in understanding content emerged in discussions following gameplay but were not immediately apparent. Participants expressed mixed views about relational functions of human support. Some participants reported that the game was better than meeting with a person because “*the game always listens to you.”* Others felt that it was important to meet with someone because they “*understand what you say.”* Participants who had previously received a face-to-face counseling intervention suggested that the game may be sufficient on its own for some participants, but others with more severe problems might also require counselor support. The human support for the game was subsequently conceptualized to offer both instruction and personalized support at onboarding and completion of the game, with the counselor available to help when needed throughout the rest of the intervention.

#### Managing Risk

Service provider FGDs highlighted concerns about ensuring appropriate provisions for risk assessment and management within the intervention. In response, a mood rating system was incorporated into the digital login procedure. This was linked to a risk assessment question that would be triggered for participants who reported very low mood. As the game was designed to work offline, a real-time alert was not possible. Instead, a positive response to the risk assessment question diverted the participant to a locked screen containing a message about approaching the supporting school counselor. A counselor would then undertake further assessment and management, after which the participant would potentially resume the intervention by the counselor using a code to unlock the screen.

#### Group Delivery

Participants in user-testing sessions were not specifically concerned about the prospect of peers knowing about their engagement with the game, although concerns were voiced about other participants observing their gameplay. Some participants expressed that group sessions may be advantageous as a way to encourage a sense of togetherness among intervention users. It was suggested private gameplay could still be achieved in a group setting if participants were able to sit at a distance from each other.

## Discussion

### Principal Findings and Relevance to Prior Work

This paper describes the collaborative design and development of *POD Adventures*, a blended gamified smartphone intervention for school-going adolescents with or at risk of anxiety, depression and conduct difficulties in India. The study aimed to understand access and appropriateness of digital interventions for adolescents and identify features to optimize: engagement with the problem-solving content, developmental, and cultural acceptability and usability and contextual acceptability and feasibility of delivery.

This study found that a digital blended self-help format was acceptable to school-going adolescents in this context. The game prototype was designed to work offline and with provision of direct counselor supervision and support during school hours in response to concerns from teachers and parents about inappropriate/unguided use of smartphones, and limited internet access at schools and participants' homes. Local infrastructural limitations, particularly the lack of online functionality, limited design decisions that might harness the full range of benefits of mobile health interventions for this population, such as “on-the-go” use to facilitate practice (40, 41) or the ability to connect with other users (12, 31). However, the design allows for further development of the game to include enhanced interactivity or online functionality in the future.

The gamified and narrative formats were found to be engaging by participants. Comprehension of problem-solving concepts was improved by revisions to the game contents wherein principles were made more explicit and concrete and supported by additional explanation, illustration, and repetition.

Gamified features such as user-choice, rewards and quizzes were found to be key aspects that participants endorsed and that enhanced their enjoyment of and engagement with the game. This is a consistent finding across the existing global mobile health (mHealth) literature and suggests these features may be fairly universal components of an engaging design (31, 38, 39). In particular, design preferences in this study bear a number of similarities to SPARX (42), a CD-Rom and downloadable app-based game delivering cognitive-behavioral therapy (CBT) to adolescents for depression. SPARX is also set in three-dimensional worlds and included characters, avatars and gamification, levels, modules, and a “guide” character. Given the universality of these features, although regional translations and cultural adaptations would be necessary, it is hoped that the basic design would be acceptable across states in India.

Instructional and relationship support were found to be valued by participants. Aspects of these support functions were shared across the in-game guide and the supporting counselor. Participants also reported feeling supported by the game itself, consistent with studies from other countries examining therapeutic alliance with digital interventions (43). It is important to note, however, that throughout the intervention planning and development process, participants and service providers also highlighted limitations of pure digital self-help to meet adolescents' needs in full. The intervention was therefore designed as blended with limited support from a counselor (41, 44). This is consistent with other global research which has found that some human support increases satisfaction (45) and adherence (46) with digital interventions for mental health. Furthermore, therapeutic alliance with a remote therapist is predictive of outcome (47).

The reconceptualization of this intervention as an open-access intervention for all help-seeking students fits with a broader move toward early interventions and selective or indicated prevention for adolescent mental health problems (35, 36). It is also aligned with evidence on problem solving as an effective strategy for emotional problems (32–34) and recent research showing positive effects of skill-based digital mental health promotion interventions for young people with depression and anxiety (48).

### Strengths

A key strength of this study was the emphasis on user-centered design, following the guidance for design of complex interventions and principles of the person-based approach to intervention development (25, 26). The iterative methodology enabled participants to guide the development and provide their inputs at each stage. Sensitivity of the researchers and design team to local and cultural context, language, participant media preferences, and digital access helped focus on user needs and formed important considerations for the prototype design. These have also been described in detail in this paper as they could inform the development of other similar kinds of programmes in the future. Another important strength is the grounding of intervention contents in relevant theory, clinical evidence and past research. Participants were balanced with regard to age, grade and gender across the different activities as much as possible. The number of adolescent participants across two diverse research sites as well as service providers that consisted of both clinical psychologists and non-specialist workers also strengthened the study. Participants with previous mental health problems who had utilized counseling services in the past were also included.

### Limitations

Researcher or design team personal views may have influenced the process of development and interpretation of results. However, the relatively large number of adolescent participants and service providers who were consulted may have helped minimize bias. Parent and caregiver perspectives could not be included and would be a valuable addition to future work.

The research methods used, i.e., focus groups, co-design workshops, and user-testing have limitations. Both teacher nominated referrals and self-selection may have introduced bias. However, the number of participants was fairly large (*N* = 46 for FGDs, *N* = 22 for co-design workshops, and *N* = 50 for user-testing sessions) and ideas and feedback began to be repeated as activities progressed indicating saturation might have been reached. Working with a larger number of participants who had experienced mental health difficulties or experienced counseling may have strengthened the prototype development. Although this study took place in two different urbanized regions in India, further work would be needed to confirm the generalizability to other settings such as semi-urban or rural settings.

### Implications for Research and Practice

There are relatively few digital interventions for adolescents or in LMICs, particularly smartphones apps that have been rigorously designed and evaluated or are available in local languages (19, 49). *POD Adventures* could help fill this gap, especially in light of the challenges of delivering accessible and timely mental health support to adolescents in LMIC settings where mental health services are under-resourced and overstretched.

In India in particular, rapidly growing penetration of mobile devices, internet and social media is increasing opportunities for wider accessibility of these kinds of interventions (50). *POD Adventures* could be adapted to settings where there is increased access to smartphones amongst adolescents (51) or where it can be integrated into existing services (for example, as part of school counseling provision or as part of broader-based health promotion activities) at lower levels of the stepped care approach (5, 52).

As blended self-help intended to be delivered with some human support *POD Adventures* is potentially more cost effective and easier to disseminate (16, 45, 46, 53). In the future, this support could be delivered through teachers, older peers or non-specialists with appropriate training (54), thereby also contributing to interventions that can be delivered in settings that have limited skilled alternative interventions or approaches, especially in LMICs (55).

### Next Steps

Future plans include the evaluation of POD Adventures through a randomized controlled trial in 2020 to evaluate effectiveness for help seeking students. A key focus of this work will include understanding optimal methods of delivery, frequency, and nature of human support required and implications for scaling up in low resource settings.

## Data Availability

Additional processed raw data and information is available by emailing pattie.gonsalves@sangath.in.

## Author Contributions

PG and EH: research design and researcher. AK, TA, and YC: research activities, intervention design, and data interpretation. RS: researcher, reviewing, and revising manuscript. DM and VP: research design, reviewing, and revising manuscript.

### Conflict of Interest Statement

The authors declare that the research was conducted in the absence of any commercial or financial relationships that could be construed as a potential conflict of interest.
